# Optimization of the RNeasy Mini Kit to obtain high-quality total RNA from sessile cells of *Staphylococcus aureus*

**DOI:** 10.1590/1414-431X20154734

**Published:** 2015-11-23

**Authors:** C. O. Beltrame, M. F. Côrtes, P. T. Bandeira, A. M. S. Figueiredo

**Affiliations:** Instituto de Microbiologia Paulo de Góes, Universidade Federal do Rio de Janeiro, Rio de Janeiro, RJ, Brasil

**Keywords:** RNA preparation, MRSA, Biofilm, Sessile cells, *Staphylococcus aureus*

## Abstract

Biofilm formed by *Staphylococcus aureus* is considered an important virulence trait in the pathogenesis of infections associated with implantable medical devices. Gene expression analyses are important strategies for determining the mechanisms involved in production and regulation of biofilm. Obtaining intact RNA preparations is the first and most critical step for these studies. In this article, we describe an optimized protocol for obtaining total RNA from sessile cells of *S. aureus* using the RNeasy Mini Kit. This method essentially consists of a few steps, as follows: 1) addition of acetone-ethanol to sessile cells, 2) lysis with lysostaphin at 37°C/10 min, 3) vigorous mixing, 4) three cycles of freezing and thawing, and 5) purification of the lysate in the RNeasy column. This simple pre-kit procedure yields high-quality total RNA from planktonic and sessile cells of *S. aureus*.

## Introduction

Formation of biofilm on biotic and abiotic surfaces is an important virulence feature of a number of medically relevant microorganisms, including *Staphylococcus aureus*. The ability to develop biofilms is critical for establishing medical device-related infections, which contribute to increased morbidity, mortality, and healthcare costs (1,2). Bacteria in the biofilm environment (sessile cells) display differential gene expression compared with free-living (planktonic) cells (3-36). The biofilm structure is formed in distinct steps, including initial attachment, maturation, and detachment (7,8). Studies have shown that polysaccharide intercellular adhesin is an important component affecting the maturation of *S. aureus* biofilms, mainly in *mecA*susceptible isolates (9). However, in methicillin-resistant *S. aureus* (MRSA), polysaccharide intercellular adhesin-independent biofilm appears to be the most common type of biofilm produced by these isolates (10-12). In addition to extracellular DNA, a number of different proteins are associated with *ica*-independent formation and accumulation of biofilm, including FnBPA and FNBPB, Spa, SasG, and more recently, PBP2a (11-16).

Although there has been some progress, the complete mechanisms involved in attachment, maturation, and detachment of biofilm in *S*. *aureus* remain undefined (11,12,16,17). In addition, few studies on biofilm gene regulation (18,19) and global gene expression of *S*. *aureus*, under the biofilm condition (4,), have been published. Messenger RNA has been increasingly used to understand the molecular mechanisms involved in modulation of biofilm. The inherent difficulty in preparing good-quality RNA from staphylococcal biofilm may be one of the reasons that limit development of such studies (23). The success of any RNA-based analysis depends on the amount, purity, and integrity of the RNA obtained (24) because these parameters may impair RNA quantification. Consequently, this influences the results from gene expression experiments. Isolation of RNA from bacterial biofilms is normally challenging because of the polymeric nature of the biofilm matrix that makes it difficult to disrupt cells within this structure by standard methods. In addition, accumulated macromolecules, such as extracellular DNA, may also clog the purification columns (23,). Recently, Atshan and colleagues tested different commercial kits to obtain total biofilm RNA from the *S. aureus* reference strain, ATCC 35556, including the RNeasy Mini Kit (Qiagen, Germany), NucleoSpin RNAII (Macherey Nagel, Germany), InnuREP RNA Mini (Jena, Germany), Trizol (Invitrogen, USA), and the MasterPure RNA Purification Kit (Epicentre Biotechnologies, USA). None of the commercial kits that were tested by these authors provided high RNA yields and they proposed a new method based on phenol extraction (23). However, phenol is corrosive (can cause severe chemical burns) and toxic. Therefore, the use of this reagent can be unsafe and should be avoided in a laboratory whenever possible.

Similarly, in our experiments, the RNA preparations obtained from sessile cells of MRSA using the RNeasy Mini Kit (Qiagen), following the protocol suggested by the manufacturer, were mostly unacceptable. However, this kit produced good-quality total RNA from planktonic cells of the same isolates. The Mini Kit is the most common commercial system used to prepare total RNA from *S. aureus*(28,29). Therefore, we designed a simple optimized protocol using the RNeasy Mini Kit that ensures high-quality RNA preparations from planktonic and sessile cells of MRSA, compatible with experiments that require integrity and good quantity of this molecule. We describe this protocol in this article.

## Material and Methods

### Bacterial isolates

The MRSA isolate BMB9393 (ST239-SCC*mec*III), which exhibits strong accumulation of biofilm (28), was used for the majority of the experiments and belongs to our laboratory collection. In addition, we also tested the biofilm producer methicillin-susceptible *S. aureus* (MSSA) isolate HC474 (28). For gene expression analyses, we included RNA obtained from the MRSA isolate GV69 (ST239-SCC*mec*III), an *agr*-dysfunctional isolate from our laboratory collection (28). The isolate USA300-0114 (received from Paul Dunman, University of Nebraska Medical Center, USA) was used as a calibrator in real-time quantitative reverse transcription-polymerase chain reaction (RT-qPCR). The strain RN4220 was used for the experiments of hemolytic activity and was a gift from Richard Novick (Skirball Institute of Biomolecular Medicine, USA). This study was approved by the Human Research Ethics Committee of the Hospital Clementino Fraga Filho, Universidade Federal do Rio de Janeiro (#136/07).

### Free-living bacterial culture

Bacterial cells were grown in trypticase soy broth (TSB; Becton, Dickinson and Company, France) at 37°C, in a shaker at 250 rpm. Aliquots were collected at the absorbance value of 0.25 (OD_600_) and at 18 h of incubation (OD_600_=4.5). The colony forming units (CFU) were determined by plating dilutions of the bacterial growth in trypticase soy agar (TSA; Becton, Dickinson and Company), after vigorous mixing.

### Formation of biofilm and collection of sessile cells

Five colonies were picked from a fresh culture on TSB plates and inoculated in 2 ml of TSB supplemented with 1% glucose (wt/vol; Sigma-Aldrich, USA). The culture was incubated at 37°C for 18 h, under shaking at 250 rpm, and diluted 1:100 in the same broth. A volume of 200 µL was placed into each well of the 96-well polystyrene microtiter plate (Nunclon; Nunc A/S, Denmark). The plate was incubated at 37°C for 20 h. After this time, the liquid culture was removed and the biofilm was washed once with RNase-free water. Subsequently, TSE buffer (20 mM Tris-HCl, pH 7.6, containing 10 mM EDTA, pH 8.0, 50 mM NaCl, and 20% wt/vol sucrose) was added to the wells, the sessile cells were dispersed with the aid of sterile toothpicks, and the CFU were determined by plating dilutions of the cell suspension in TSA, after vigorous mixing.

### Bacterial lysis and RNA preparation using the RNeasy Mini Kit

Approximately 10^8^-10^9^ bacterial cells were treated with 100 µg/mL lysostaphin (500 U/mg; Sigma-Aldrich) at 37°C for 10 min. After this incubation, the protoplasts were used for RNA isolation with the RNeasy Mini Kit, following recommendation of the manufacturer for RNA preparation from bacterial cells (Qiagen). In another lysis protocol, bacterial cells (10^8^-10^9^ CFU/700 µL) were transferred to 2 mL lysing matrix B tubes (MP Biomedicals, USA) and disrupted at the reciprocating device FastPrep FP120 (MP Biomedicals), using the following settings: 5.0 m/s for 20 s (first pass), 5-min rest period on ice, and 4.5 m/s for 20 s (second pass). After centrifugation at 9,000 *g* at 4°C for 5 min, the supernatant was used for RNA isolation with the RNeasy Mini Kit, according to the manufacturer’s recommendations. More than three independent experiments were performed for each procedure.

### Optimized method for RNA isolation from sessile cells using the RNeasy Mini Kit

To improve the quality and quantity of the total RNA that was obtained from sessile cells of *S. aureus*, we modified the protocol for RNA preparation by using sheared whole-cell lysate coupled to RNA isolation using the RNeasy Mini Kit. The sheared whole-cell lysate method was based on that described by Kornblum et al. (30) with some modifications, as follows. Bacterial cells were collected as described above, except that the TSB culture and the sessile cells that were detached from biofilms were treated with 1 volume of acetone-ethanol (1:1). The cells were left for 20 min in an ice bath or stored at −80°C. On the day of the experiment, the cell suspension in acetone-ethanol was washed once in TSE, resuspended in the same buffer, and adjusted to contain 10^8^-10^9^ CFU/700 µL. After enzymatic or mechanical lyses, as described above, 350 µL of RLP buffer (Qiagen) containing 14.4 M 2-mercaptoethanol (Amershan Biosciences, Germany) was added to each 200 µL of the cell lysate. The lysate was then placed in a microtube mixer for 30 min at 4000 rpm (Marconi, Brazil) at room temperature. Subsequently, the material was quickly frozen (ethanol-dry ice) and thawed (water bath at 60°C) three times. Finally, the RNA was purified using the RNeasy Mini Kit, following the manufacturer's specifications for bacterial cells.

### DNase I treatment

To ensure that the total RNA from the sessile cells was free of DNA, the first treatment with DNase I was performed during the RNA clean-up with the RNeasy Mini Kit, following the manufacturer’s instructions (Qiagen). A second treatment with DNase I was performed after eluting the purified RNA from the column, as recommended by the manufacturer (Invitrogen). The RNA preparation was stored at −80°C.

### RNA quantification

Total RNA was quantified using the NanoDrop 1000TM (Thermo Scientific, USA).

### Gel electrophoresis

The integrity of total RNA was initially assessed by visualization of the 23S/16S banding pattern using 1.2% agarose gel electrophoresis in 1×TAE (20 mM Tris acetate, 0.5 mM EDTA, pH 8.0) run at 110 V for 50 min. The gel was treated with ethidium bromide and visualized in a gel capture system (DNR Bio-Imaging System, Israel).

### Real-time qRT-PCR

To further analyze the quality and stability of the RNA preparations, 0.1 ng of total RNA was reverse transcribed, and cDNA was amplified using the Power SYBR¯ Green RNA-to-CTTM 1-Step Kit (Applied Biosystems, USA), according to the manufacturer's instructions. Real-time qRT-PCR was performed to relatively quantify mRNA of well-known virulence genes of *S. aureus*, the *agr*RNAIII-downregulated *spa* (encoding protein A), and the *agr*RNAIII-upregulated *psmα3* (encoding phenol soluble modulin α3). In addition, levels of RNAIII, the effector molecule of the Agr virulence regulator system, were also determined. The gene encoding for 16S rRNA was used as a reference. The reactions were standardized to a total reaction volume of 20 μL and cycling conditions for all primers were as follows: an initial cycle at 48°C for 30 min (for obtaining cDNA); and a denaturation step at 95°C for 10 min and 35 cycles at 95°C for 30 s, 55°C for 45 s, and 72°C for 45 s (for cDNA amplification). The experiment was performed using the StepOne Real-Time PCR System (Applied Biosystems). To ensure the absence of genomic DNA, a negative control was included, without reverse transcriptase. The cycle threshold (Ct) of each gene amplification was determined using the standard parameters of the software. Melting curves were evaluated to ensure the absence of primer-dimer formation and unspecific products. Relative quantification of the transcripts was determined by using the ΔΔCt method as described in the StepOne and StepOnePlus Real Time PCR Systems Getting Started Guide (Applied Biosystems). Data analysis was based on two independent experiments with triplicates, using StepOne Software 2.2 (Applied Biosystems). Table 1 lists the primers that were used in the study.

**Table:**
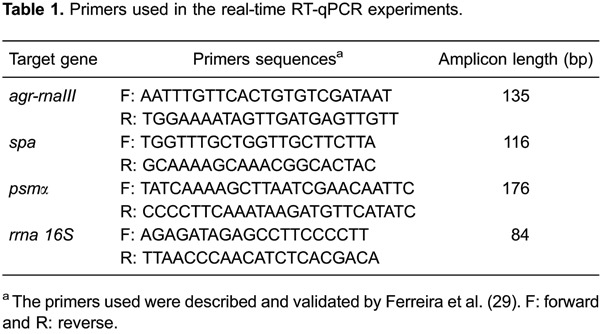

### Hemolytic activity

The *hld* gene, encoding for δ-hemolysin (Hld), is codified within the *rnaIII* region. Consequently, detection of δ-hemolysin is an indication of *agr* expression. To correlate Hld activity with the results obtained in the gene expression experiments, the biofilm producers BMB9393 (*agr*-functional) and GV69 (*agr*-dysfunctional) were also tested for hemolytic activity on plates containing a blood agar base with 5% defibrinated sheep blood (Plast Labor, Brazil), as previously described (31).

### Enrichment of mRNA

To further insure the quality of bacterial mRNA obtained by the optimized protocol, total RNA preparation from sessile cells was enriched using the MICROB*Express*™ Bacterial mRNA Enrichment Kit (Ambion, Life Technologies, USA), following the specifications of the manufacturer. To determine the concentration, integrity of the mRNA, and percentage of rRNA contamination, the material was analyzed using the Agilent BioAnalyzer with the 2100 Expert mRNA Pico Chip, following the manufacturer’s recommendations (Agilent Technologies, USA).

## Results and Discussion

### Quality control of the RNeasy Mini Kit

**Figure 1:**
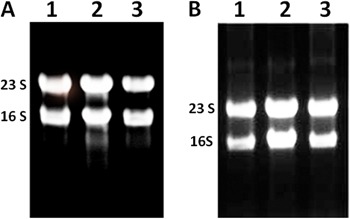
RNA that was obtained from the methicillin-resistant *S. aureus* (MRSA) isolate BMB9393, which was grown in the logarithmic (*A*) or stationary (*B*) phase using mechanical (*lane 1*), enzymatic (*lane 2*), and sheared whole-cell lyses (*lane 3*).

Using the lysis protocols that are described above in the Material and Methods section, we determined if the RNeasy Mini Kit was working properly when total RNA was obtained from planktonic staphylococcal cells. We carried out RNA preparations from logarithmic- and stationary-phase MRSA cells. We obtained high-quality RNA when logarithmic- or stationary-phase free-living cells were used, independently of the protocol that was chosen for lysis (Figure 1).

### Preparation of total RNA from sessile cells using the RNeasy Mini Kit

**Figure 2:**
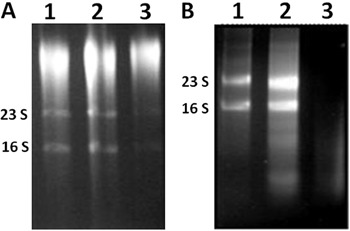
Gel electrophoresis of total RNA that was obtained from sessile cells of the methicillin-resistant *S. aureus* (MRSA) isolate BMB9393. *A*, Mechanical and *B*, enzymatic lyses.

When enzymatic (Figure 2A) or mechanical lysis (Figure 2B) was used to obtain total RNA from sessile cells, the results were inconsistent, since they did not always yield good-quality RNA. On the other hand, when the RNA prepared by these methods was not degraded, the amount obtained (50-150 ng/µL) was insufficient for most gene expression experiments (e.g., RNA microarrays or mRNA enrichment).

### Sheared whole-cell lysis coupled to the RNeasy Mini Kit

**Figure 3:**
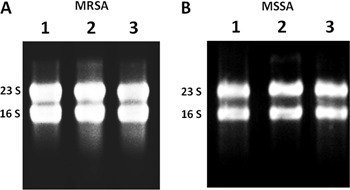
Gel electrophoresis of total RNA that was obtained from sessile cells with sheared whole-cell lysis coupled to the RNeasy Mini Kit. *Lanes 1*, *2*, and *3*represent three independent experiments using the methicillin-resistant *S. aureus* (MRSA) isolate BMB9393 (*A*) and the methicillin-susceptible *S. aureus* (MSSA) isolate HC474 (*B*).

Treatment of free-living or sessile bacterial cells with acetone-ethanol 1:1 and vigorous mixing, combined with three cycles of freeze/thawing after lysostaphin treatment, resulted in increased quantity and high-quality RNA. These results were visualized by ribosomal RNA band patterns after total RNA separation using gel electrophoresis (Figure 3A,B). The amount of RNA that was obtained from sessile cells for MRSA and MSSA varied from 500-700 ng/µL. This concentration could also be increased by loading the same RNeasy column with two lysates, but eluting the RNA in 40 µL of H_2_O.

### Evaluation of RNA quality for gene expression experiments

**Figure 4:**
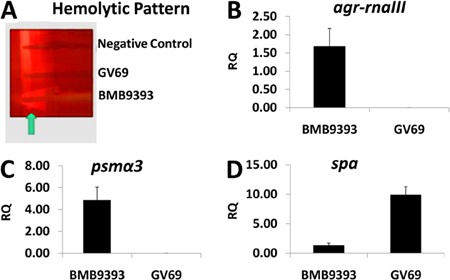
*A*, Hemolytic pattern of the methicillin-resistant *S. aureus* (MRSA) isolates BMB9393 and GV69. The arrow indicates the arrow-tip-like zone of δ-hemolysin activity on sheep blood agar. *B*, *C*, and *D*, Transcriptional levels of virulence-associated genes determined by real-time RT-qPCR, using the ΔΔCT comparative method. *B*, *agr*-*rna*III, *C*, *psmα3*, and *D*, *spa*. Total RNA was prepared from stationary-phase cells. The USA300 isolate was used as a calibrator. RQ: relative quantity.

To further evaluate the quality and integrity of the RNA preparations, we performed some gene expression experiments using real-time qRT-PCR, which is considered the gold standard technique for validation (e.g., genome-wide expression analyses) (31). We chose to analyze the transcriptional levels of some virulence genes of *S. aureus* for which the expression patterns are well known, and its regulation is under control of the main *S. aureus*quorum-sensing system, Agr (32). For these analyses, we obtained total RNA from sessile cells of two *S. aureus* isolates (*agr*-functional isolate BMB9393 and the naturally *agr*-dysfunctional GV69), using sheared whole-cell lysis coupled to the RNeasy Mini Kit (28). The hemolytic patterns of BMB9393 and GV69 are shown in Figure 4A. We observed a hemolytic pattern formed by synergism between β- and δ-hemolysins in the *agr*-functional isolate (BMB9393). This finding was due to expression of the *hld* gene codified in the region of *agr*-*rnaIII* (32). We did not observe hemolytic activity by the *agr*-dysfunctional isolate GV69, confirming the Agr impairment. As expected, the expression of *agr*-RNAIII was higher in BMB9393 compared with that obtained for GV69 (Figure 4B). Additionally, the RNAIII-upregulated *psmα* gene was more highly expressed by sessile cells of the BMB9393 isolate compared with GV69 (Figure 4C). In accordance with RNAIII-downregulation of *spa*, the transcriptional level of this gene was higher in the *agr*-dysfunctional isolate than in the *agr*-functional isolate (Figure 4D).

**Figure 5:**
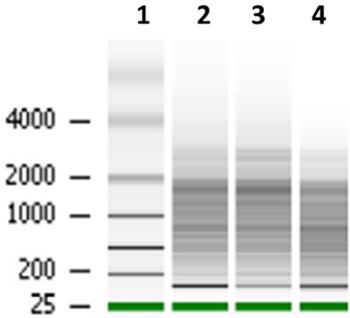
Gel image from the Agilent 2100 Bioanalyzer of enriched mRNA preparation. *Lane 1*, ladder; *lanes 2*-*4*, enriched mRNA preparation that was obtained from sessile cells of the BMB9393 isolate using total RNA from the optimized protocol.

The quality and integrity of the RNA preparations using the modified protocol, were futher accessed using the Agilent 2100 Bioanalyzer. The gel images showed no mRNA smearing, indicating that there was no degradation of this molecule (Figure 5). In addition, 94-99% of the ribosomal RNA was removed from this RNA preparation, consistent with what is expected for this kit.

In conclusion, we present a simple and optimized procedure for total RNA preparation from sessile cells of MSSA and MRSA. Our modified protocol provided high-quality RNA, when coupled to the RNeasy Mini Kit. Good-quality RNA obtained from sessile cells is one of the main obstacles to overcome so that reliable studies (e.g., for global gene expression using RNA from sessile cells) can be performed with accuracy. The advantage of the proposed protocol is that it avoids the use of phenol, as proposed by Atshan et al. (23). This enables cost-saving and less chemical toxicity. Additionally, we used the RNase Mini Kit, which is commonly used for obtaining total RNA from *S. aureus* cells (29-31).

Previous studies have demonstrated that there is a good correlation between *S. aureus* biofilms that are developed *in vitro* and *in vivo* (29). Indeed, staphylococcal fibronectin-binding protein, a major biofilm-associated molecule, can support bacterial adhesion to abiotic surfaces, in addition to promoting biofilm development *in vivo* by binding to the host fibronectin (11). Although we did not test this methodology for obtaining RNA from *S. aureus*biofilms that develop during the course of an infection, problems related to a low number of bacterial cells and contamination with eukaryotic RNA can be critical. However, these difficulties could possibly be overcome by growing sessile cells collected from an *in vivo* model, in an enriched culture media for approximately 10 generations, to maintain the *in vivo* adapted stage (33).
